# The Treatment of High Blood-Pressure

**Published:** 1907-04-27

**Authors:** 


### THE TREATMENT OF HIGH BLOOD-PRESSURE.

At a recent meeting of the Therapeutical Society Dr. George Oliver gave some results of his clinical observations on the management, by diet, baths, electricity, and internal remedies, of a persistent rise in the arterial pressure. He pointed out the value of a non-stimulating, and especially of a lacto-vegetarian and salt-free diet; of a minimum proportion of calcium salts in drinking water; of warm massage douching; of saline and carbonic-acid baths; of D'Arsovalisation and the electric light and ozone baths; of warm equable climates (Jamaica, India, and Egypt); of evacuant treatment; of asepsis of the bowels; and of depressor remedies. He indicated some practical suggestions furnished by physiology in the selection of depressor remedies for more or less continuous use, and stated his favourable experience of the benzene derivatives. At the same time he showed that our control is limited to cases in which the rise is moderate and the vaso-motor play of the peripheral arteries is not seriously impaired. In the discussion which followed, Sir Lauder Brunton said that with perfect rest, vegetarian diet, and the steady use of nitrites, even cases of exceedingly high tension might be successfully treated. A salt-free diet might be useful, but it could not be continued for a length of time, as it is so irksome, although salt might to a considerable extent be replaced by sugar in the dietary. The modern treatment of high tension by nitrites and nitrates had been recommended by Lord Bacon. The nitre Bacon employed probably contained a quantity of nitrites; and blood-letting was a remedy which had fallen too much into disuse, and might be employed again with advantage. Dr. Herbert French said cases of very high blood pressure occurred even amongst those who were at the same time vegetarians, non-smokers, and life-long abstainers. Dr. Oliver, in reply, agreed that rest in bed would diminish the high blood-pressure even in the worst cases, but this was owing to a diminished call upon the cardiac end of the circulation. In those who were obliged to work, the ventricle-factor could not be diminished, and such cases seldom reacted to depressor drugs so long as the patient remained up and about.

